# Beneficial Diets and Pancreatic Cancer: Molecular Mechanisms and Clinical Practice

**DOI:** 10.3389/fonc.2021.630972

**Published:** 2021-05-28

**Authors:** Yang Zhang, Tao Zhang, Wenbo Yang, Hongze Chen, Xinglong Geng, Guanqun Li, Hua Chen, Yongwei Wang, Le Li, Bei Sun

**Affiliations:** ^1^ Department of Pancreatic and Biliary Surgery, The First Affiliated Hospital of Harbin Medical University, Harbin, China; ^2^ Key Laboratory of Hepatosplenic Surgery, Ministry of Education, Harbin, China

**Keywords:** diet, ketogenic diet, low-sugar diet, Mediterranean diet, pancreatic cancer

## Abstract

Pancreatic cancer (PC) is a malignant tumor with high invasiveness, easy metastatic ability, and chemoresistance. Patients with PC have an extremely low survival rate due to the difficulty in early diagnosis. It is estimated that nearly 90% of PC cases are caused by environmental risk factors. Approximately 50% of PC cases are induced by an unhealthy diet, which can be avoided. Given this large attribution to diet, numerous studies have assessed the relationship between various dietary factors and PC. This article reviews three beneficial diets: a ketogenic diet (KD), a Mediterranean diet (MD), and a low-sugar diet. Their composition and impact mechanism are summarized and discussed. The associations between these three diets and PC were analyzed, and we aimed to provide more help and new insights for the prevention and treatment of PC.

## Introduction

**Figure 1:**
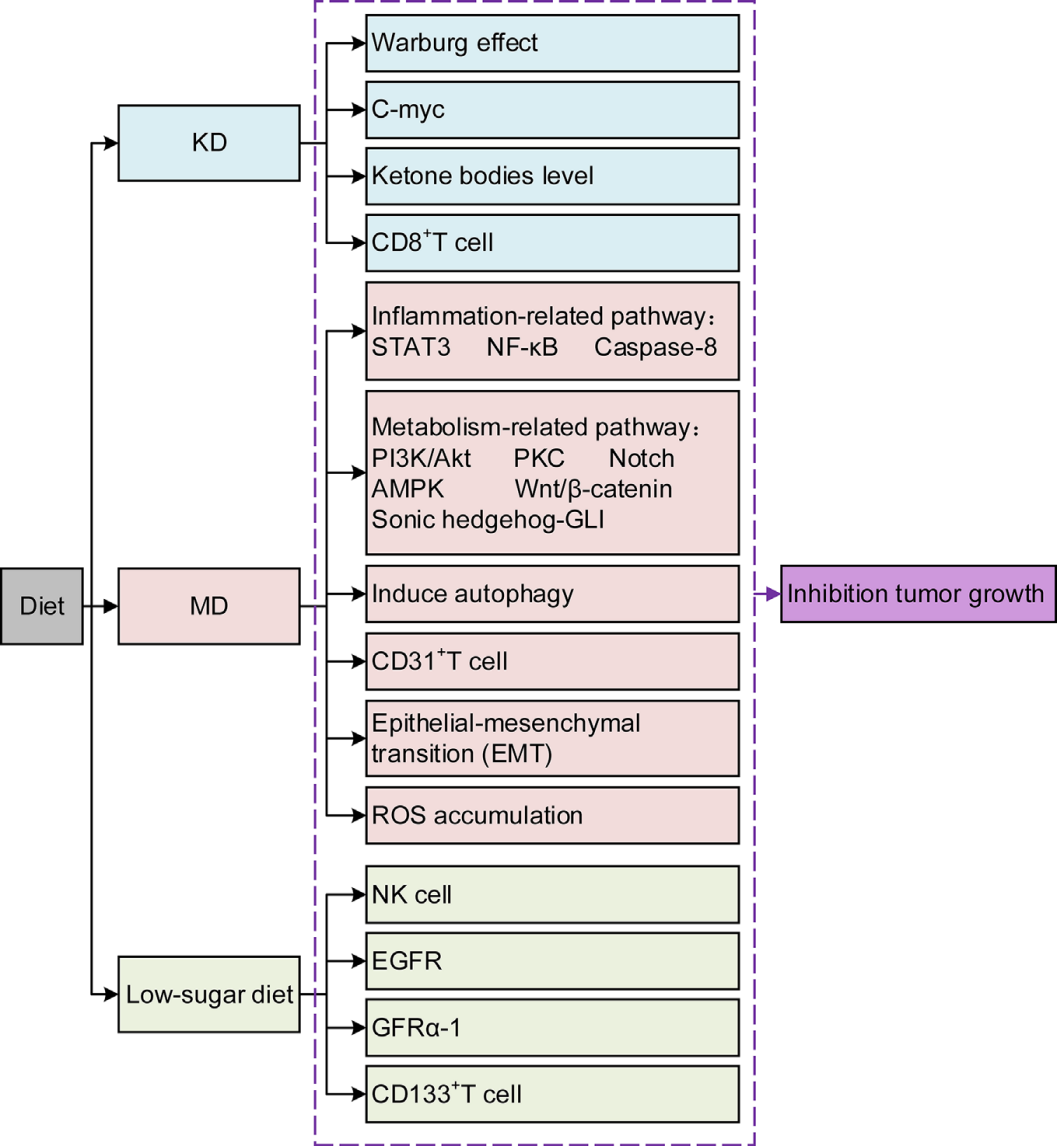
The molecular mechanism between a beneficial diet and pancreatic cancer.

Pancreatic cancer (PC) is known for its insidious onset, invasive fast-growing nature, and poor prognosis (1). Although radical resection is currently the primary therapeutic option, most patients miss the appropriate time due to its unspecific early clinical manifestation. Although great progress has been made in radiotherapy (RT), chemotherapy, and immunotherapy in recent years, the desired results have not yet been achieved (2). Avoiding high-risk factors is the first and most crucial step to change its incidence. Current evidence suggests that up to one-third of cancer deaths can be prevented by reducing risk factors, and an unhealthy diet is one of the most crucial factors (3). Dietary composition affects tumor growth and progression and creates potential synergies or antagonisms between new or existing therapeutics (4). Diet can affect tumor growth through various mechanisms that alter cancer cell metabolism **(** **Figure 1** **)**. The components within the diet could even improve the prognosis by affecting drug efficacy and resistance (5). Research on the relationship between dietary composition and cancer risk is becoming increasingly crucial. This review stresses several different kinds of typical diets, and the effects of some components within the diet are analyzed in PC treatment. The aim is to deepen the knowledge about the role of diets in PC and the underlying mechanisms, which provides evidence for further developing PC prevention strategies. Second, it is hoped that this study can fill the gaps in the treatment methods of PC and improve the treatment effect and patient survival rate. Finally, we aim to promote multidisciplinary prospective research to advance the field.

## The Ketogenic Diet (KD)

There is no precise definition of the KD. Thus, many studies have defined it as any diet that leads to an increase in blood ketones (6), for example, diets in which more than 50% of total calories are from fat (7). However, the KD is generally accepted to be characterized by high fat, moderate protein, and very low carbohydrate composition. The classical KD contains four parts fat, one part carbohydrate, and one part protein (4:1:1) (8). It delivers 90% of its calories from fat, 8% from protein, and only 2% from carbohydrates. However, the KD used in the clinical setting has a ratio of fat to carbohydrate and fat to protein of at least 2:1 and 3:1 (9). The development of the KD has been relatively completed thus far. All-liquid and parenteral KDs have been studied (10), thus, the diet is often started on an outpatient basis (11).

The KD is regarded as a metabolic therapy widely used in the treatment of epilepsy (12). Recent studies have confirmed the therapeutic potential of the KD in many pathological conditions over the last decade, including diabetes, polycystic ovary syndrome, acne, neurological diseases, malignant tumors, and the amelioration of respiratory and cardiovascular disease risk factors (13, 14). A growing number of preclinical studies suggest that the KD as a dietary intervention is a potent anticancer therapy (15).

Various investigators have used the term “therapeutic ketosis”, which implies the achievement of plasma ketone body levels in the 2-7 mmol/L range, comparable to concentrations found in subjects maintained on various KDs (16). The KD leads to an increase in ketone bodies without restricting energy intake, a clear advantage in the cancer setting (17). The KD suppresses the Warburg effect by inducing tumor starvation, which is generally considered its anticancer mechanism (18). The Warburg effect is characterized as mainly adopting glycolysis as an energy source to maintain tumor cell growth and biosynthesis in cancers (19). Glucose dependency and lactate production are recognized as two key features related to the aggressive capacity and invasive potential of cancer cells (20). Normal cells readily use ketones as an alternate energy source and induce a shift in cellular energy supply from glucose to fatty acids and ketones to protect against hypoglycemia when ketone body levels rise (21). Cancer cells are incapable of ketone body metabolism due to their acquired metabolic inflexibility (22). As a result, ketone bodies cannot be consumed, and tumor development is inhibited (23). Many studies have confirmed that the KD has a positive effect on various types of cancer and could retard the progression of gastric cancer, prostate cancer, and brain cancer in mouse models (24, 25). In addition, the KD was found to eliminate tumor growth by inhibiting angiogenesis and reducing tumor volume in preclinical trials (26). This ability was linked to the reduction in glucose availability, insulin, and circulating insulin-like growth factor (IGF)-1 levels (27, 28). Ketosis has been confirmed to be inversely associated with serum insulin levels and correlated with stable disease or partial remission (29).

Husain et al. (30) confirmed that the KD could decrease the activation of natural killer cells, improve the numbers of myeloid-derived suppressor cells (MDSCs) in a PC mouse model, and enhance the host immune response to tumor cells. This suggested that several mechanisms could support the effectiveness of the KD in cancer treatment, far beyond the originally proposed inhibition of glucose/insulin signaling, including oxidative stress, mitochondrial metabolism, and inflammation (31). The increased oxidative stress and reactive oxygen species (ROS) production are attributable to mitochondrial damage (32). In addition, chronic inflammation from sustained hyperglycemia also represents a major source of ROS production in tumors (33). Stafford et al. (34) reported that the KD reduced the rate of tumor growth and prolonged survival with reduced ROS production in cancer cells. Ketosis protects against oxidative stress in healthy tissues by simultaneously enhancing endogenous antioxidant capacity and decreasing ROS production (35). Moreover, cancer cells are inefficient in metabolizing toxic substances (28). These factors allowed the KD to selectively inhibit metabolism in cancer cells but not in normal cells.

Surgery remains the primary treatment for PC, and radical tumor resection can considerably reduce the risk of cancer recurrence and increase the 5-year survival rate (36). However, patients who undergo pancreatectomy are more susceptible to malnourishment and weight loss due to complications, such as pancreatic fistula and delayed gastric emptying (37). It has been suggested that the KD improves meal compliance, satisfaction, and the energy intake rate in post-pancreatectomy patients without increasing complications of the digestive system. It is a safe way to increase energy and nutrient intake in pancreato-biliary cancer patients after surgery (38, 39). Unfortunately, only 20-30% of PC patients are candidates for surgical resection, as most are diagnosed with locally advanced PC or metastatic PC (40). As a result, chemotherapy must be conferred as a survival advantage for those patients. Recent guidelines recommend gemcitabine monotherapy or gemcitabine-based combination therapies; however, even the FOLFIRINOX (5-fluorouracil, folinic acid [leucovorin], irinotecan, and oxaliplatin) regimen is considered as an option for some patients (41, 42). RT, chemotherapy, and current nonsurgical standard therapies for cancer therapies share a common mechanism, which is to kill cancer cells by enhancing the production of ROS (43). Therefore, the application of a KD during treatment would selectively enhance tumor cell versus normal cell sensitivity to RT and chemotherapy by inducing oxidative stress (44).

**Figure 2:**
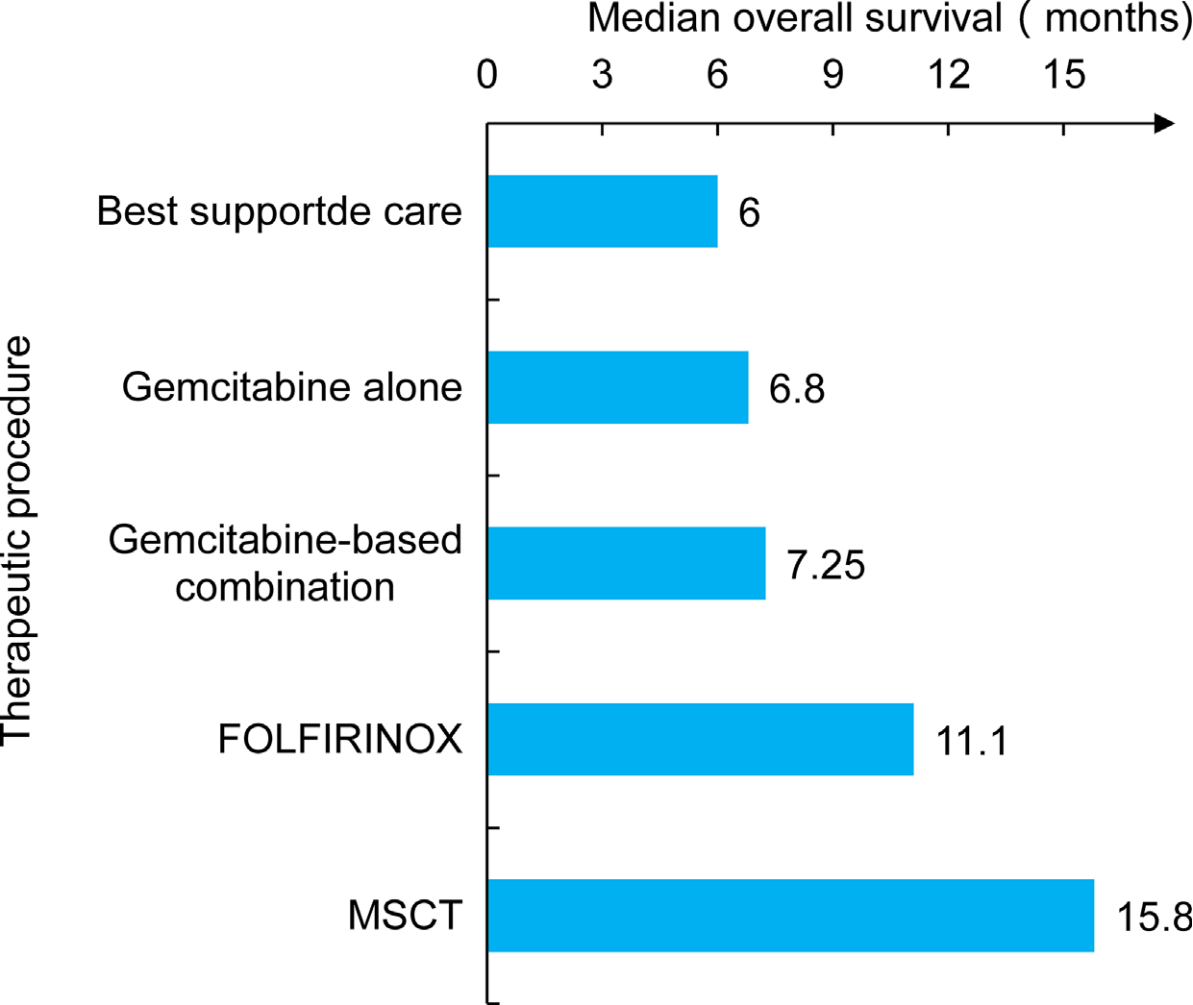
The study compared several treatment modalities, including earlier traditional care and chemotherapy. The patient’s survival time was significantly prolonged when combined chemotherapy with metabolic support and the KD (MSCT).

Metabolically supported chemotherapy (MSCT) is a novel approach targeting the dysregulated energy mechanism of tumor cells and has been generally combined with KD, hyperthermia (HT), and hyperbaric oxygen therapy (HBOT) in advanced PC patients (45, 46). The effects of MSCT (either gemcitabine based or FOLFIRINOX regimen administered concomitantly with induced hypoglycemia) plus the KD, HT, and HBOT combination have been investigated in a clinical trial. A total of 25 metastatic pancreatic ductal adenocarcinoma (PDAC) patients were enrolled, and past data were compared. The results showed that the overall median survival rate of patients receiving gemcitabine therapy alone was approximately 6.8 months, and that of the FOLFIRINOX group was 11.1 months. Encouragingly, the combination of the KD with MSCT achieved an overall median survival rate of 15.8 months and a progression-free survival rate of 12.9 months **(** **Figure 2** **)** (47). Talib et al. (48) demonstrated that a combination of the KD with melatonin could successfully inhibit cisplatin- and vincristine-resistant breast cancer, which indicates that the KD may improve the treatment effect of drug-resistant patients. In addition, there was evidence of increased survival in mice grafted with high-grade glioma, lung, or PC cells when mice received a KD in association with RT (49, 50). A phase I clinical trial of the KD and PC patients was also conducted, but it was terminated due to suboptimal oral KD compliance and poor tolerance (51). Another trial enrolled 70 cancer patients, half of whom received a KD during RT. The results revealed that the KD improves patient tolerance by improving muscle mass against cachexia (52). This indicated that the KD might be of unexpected value for PC patients at high risk of weight loss and receiving RT and chemotherapy.

As reported in most preclinical studies, the process of cancer-induced cachexia can be reversed by a KD (53, 54). The incidence of cachexia in PC is 80%, characterized as a complex metabolic syndrome associated with the advanced disease stages (55). Surendra et al. (56) demonstrated that reversal of the metabolic syndrome induced by ketone bodies might be related to the levels of c-Myc in PC. The ketone body-reduced c-Myc expression suggests that the KD is beneficial to PC. Greene et al. (57) found that a KD decreased body mass without adverse effects on skeletal muscle and muscle strength. In addition, Andrew et al. (58) demonstrated that R/S1,3‐butanediol acetoacetate diester, a novel synthetic ketone diester, attenuates tumor burden indices by diminishing anorexia, altering systemic metabolism, and mitigating catabolism in a cancer‐independent multifactorial inflammation-induced atrophy environment. This indicated that ketone bodies participate in anticatabolic effects by eliminating multifactorial atrophy. Nakamura et al. (59) postulated that elevated blood ketone levels might have antitumor effects by promoting the maintenance of body weight and muscle mass, leading to a reduction in inflammation. KD treatment of cachexia aims to maintain skeletal muscle mass and physical performance, avoid interruption of scheduled anticancer treatments, and improve quality of life (60).

The KD might indirectly affect the efficacy of immunotherapy by affecting the metabolism of the host. Flint et al. (61) indicated that the metabolism of calorie-deficient mice would be altered to develop hypoketonemia, which triggered a marked rise in glucocorticoid levels, affecting the effectiveness of immunotherapy in PDAC. They conducted further studies on this view and found that the increased level of ketone bodies in PDAC mice inhibited the systemic metabolic stress response, reducing the suppression of immunotherapy (62). Another study found that glucose-dependent CD8^+^ tumor-infiltrating lymphocytes (TILs) could undergo a competitive disadvantage for nutrients that might negatively affect their immune function. However, the KD significantly reduces the expression of the inhibitory ligand PD-1 (PD-L1) on CD8^+^ T cells (63). Additionally, mice fed a KD presented lower expression of PD-L1 in tumor cells that notoriously inhibited CD8^+^ T cell activity (64). These results suggested that the KD may alter tumor-mediated T cell suppression by reducing the number of cells susceptible to the PD-1 inhibitory pathway. A recent study demonstrated that the enhancement of lipid catabolism in CD8^+^ T cells increases the efficacy of immunotherapy within a tumor microenvironment low in carbohydrates (65). Mounting evidence has highlighted that nutrient modulation also improves cancer immune surveillance so that severe immunosuppression could be avoided or even that the immune response or immune-based cancer therapies could be potentiated through patient microbiota remodeling (66). The convergence of host metabolism and antitumor immunity may offer a platform for investigations of new combination therapies.

Most experiments have shown that the KD is safe (37–39). The side effects caused by the KD, including constipation, fatigue, leg cramps, vomiting, lack of energy, and hunger, are reversible (67, 68). In addition, the KD should be low in proteins (mainly vegetable proteins) and carbohydrates (non-starchy vegetables) and high in lipids [mainly unprocessed vegetable oils rich in polyunsaturated fatty acids (PUFAs)] (16, 21, 69). It is worth noting that the classic artificial KD may be deficient in vitamins and minerals (70). Adequate supplementation of these micronutrients is essential and should be planned and monitored by physicians and qualified dieticians (71). The paleolithic ketogenic diet (PKD) is based on animal fat, meat, and offal with a fat: protein ratio of about 2:1 (72). The PKD differs from the classical KD in that it excludes food components that are not available in preagricultural times, and it supplies optimal amounts of micronutrients (73). Tóth et al. have proven that the PKD has a considerable effect on soft palate cancer, rectal cancer, glioblastoma multiforme, and cervical intraepithelial neoplasia (72–75). The researchers assume that this diet is evolutionarily advantageous for humans and has superior effectiveness compared to the KD in cancer management (72, 73). Thus, the PKD provides hopes for refractory cancer therapy and we do believe that further studies should be conducted to explore the possible mechanisms of PKD in the treatment of cancer and other chronic diseases.

In most preclinical models, the overall beneficial effects of the KD were suggested (17, 24, 25). However, most of the data took advantage of the mouse model, limiting the translation to preclinical studies (53, 54). Many clinical trials have involved only a few patients, and in some cases, a control group was not included (23, 29). A high level of heterogeneity among studies prevents the formulation of conclusions. In addition, apart from the earliest study on two pediatric oncology patients, other studies on KD were limited in follow-up duration (23). There is no clear evidence available demonstrating long-term benefits of KD, as regards hard clinical endpoints, such as survival or mortality. However, due to the special biological characteristics of PC, many therapies were not very effective. Therefore, it is currently impractical to discuss whether KD can affect the survival or mortality of PC. Researchers should focus on the effects of combining KD with other therapies, such as chemotherapy or neoadjuvant therapy. We are eager to know whether the combination could decrease the tumor stage, reduce the distant metastasis, or even relieve the side effects of such kinds of treatments. Furthermore, high-quality randomized controlled trials should be taken into account to gain more evidence for bringing KD into clinical practice.

## The Mediterranean Diet (MD)

### The Composition of the MD

**Figure 3:**
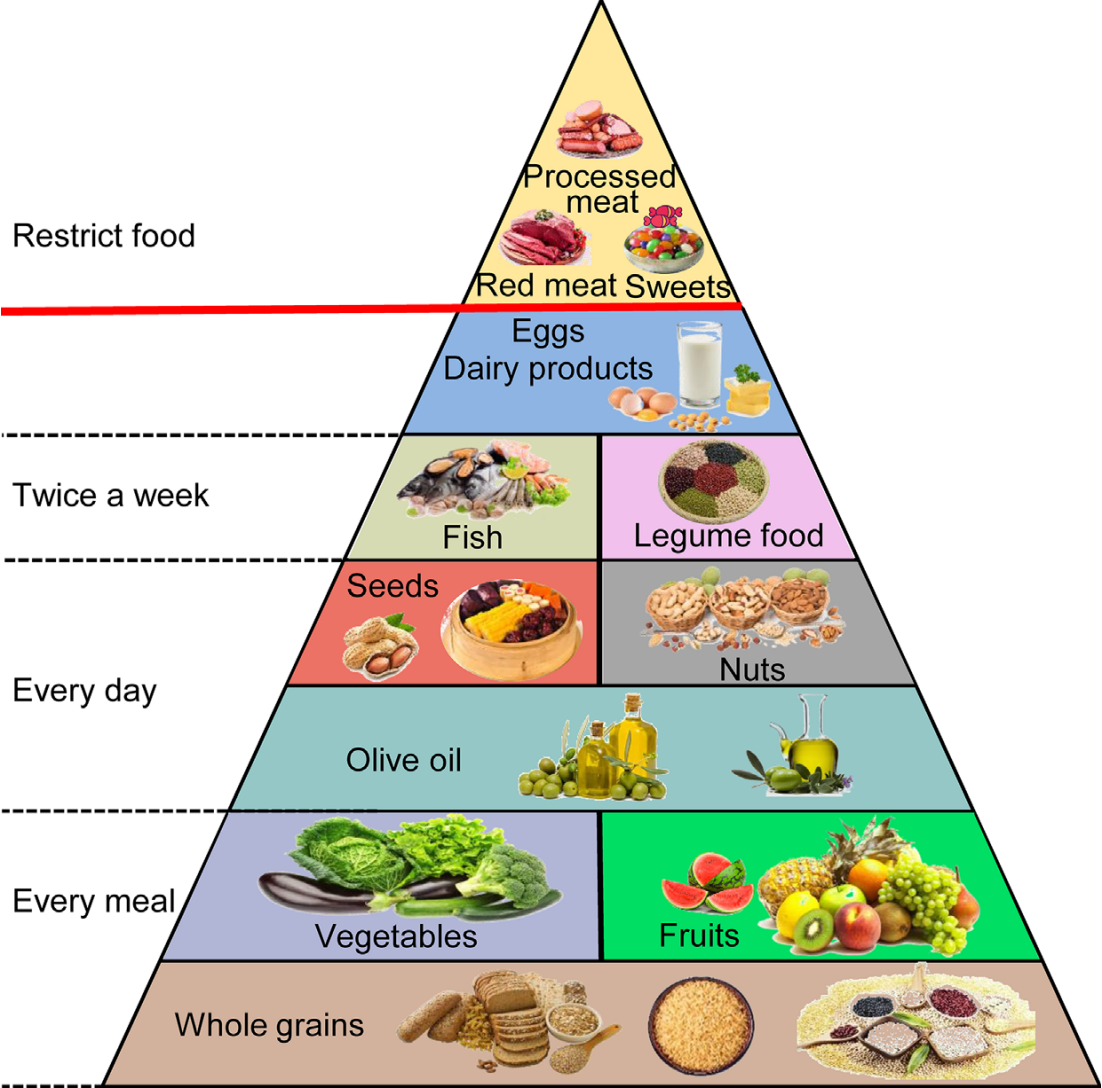
The Mediterranean diet.

The MD originates in the food cultures of ancient civilizations that developed around the Mediterranean Basin, and this term is used today to describe the traditional dietary habits of countries neighboring the Mediterranean Sea, mainly Greece and southern Italy (76, 77). Its detailed components contain fruits, vegetables, and whole grains in every meal. Olive oil, tree nuts, and seeds are consumed every day. The aim is to eat fish, seafood, and legume products at least twice a week. Meat and eggs are consumed infrequently and in small quantities, and processed meat and sweets are practically nonexistent **(** **Figure 3** **)** (78, 79). This diet contains 40% to 50% carbohydrates (approximately 80% coming from complex carbohydrates such as whole grain bread), 10% to 20% protein (particularly fish), and 30% to 40% fat (mainly from polyunsaturated ω-3 FAs) (80, 81). Higher adherence to the MD can extend lifespan and seems to have an inverse association with the risk of gastrointestinal cancers, including PC, by affecting gut microbes, hormone receptors, fat, obesity, and other aspects (82–84). However, some limitations could be found in these studies. First, the distinction between different food groups is imprecise and overlapping. For instance, the vegetable group includes beans, and the legume group contains peanuts that are often covered by the nut group. Second, many foods are not associated with the same category, although similar biologically active substances are present. It is difficult to classify them according to their specific effects (85). Therefore, we will discuss the bioactive compounds that have greater impacts on PC in the natural products in the MD rather than categorize them by the types or species.

### ω-3 FAs and PC

Many epidemiological studies have accumulated evidence regarding the effect of ω-3 PUFAs, namely, α-linolenic acid, eicosapentaenoic acid (EPA), and docosahexaenoic acid, in cardiovascular disease, metabolic syndrome, cancer, and immune system disorders (86, 87). It is estimated that the minimum human requirements are 0.2% of daily energy intake for ω-3 PUFAs (88). The typical MD recommends consuming marine fish that provide ω-3 FAs and eating vegetables, nuts, and virgin olive oil to complement derived ω-3 FAs (89). Recent studies have shown that ω-3 PUFAs inhibit cancer cell growth in colorectal cancer (CRC) and cholangiocarcinoma (90, 91). Song et al. (92) found that ω-3 PUFAs inhibited PC growth by reducing the β-catenin expression and T cell factor/lymphoid-enhancing factor reporter activity. A study found that a 5% fish oil (FO)-supplemented diet rich in ω-3 FAs significantly suppressed tumor growth by inducing ROS accumulation and caspase-8-dependent cell death (93). In addition, the accumulated EPA in the pancreas was shown to concomitantly promote autophagy and diminish its ability to induce apoptotic cell death (94). The combination of EPA with an autophagy inhibitor may be a useful strategy in increasing the therapeutic effectiveness in PC. ω-3 FAs also manifest synergistically with chemotherapeutic agents and enhance tumor radiosensitivity (95). Two clinical trials found that gemcitabine plus an ω-3-rich lipid emulsion improved the response rate and disease control rate (96) and improved PC patient prognosis (97, 98). Another study investigated whether the application of ω-3 FAs significantly prolonged the median survival time (MST) of patients (99). Hering et al. (100) demonstrated that ω-3 FAs plus gemcitabine inhibited gemcitabine-induced NF-κB activation, restored apoptosis, and reduced mortality due to gemcitabine chemoresistance. Similar to the KD, ω-3 FAs can also improve the skeletal muscle mass of PC patients against cachexia and simultaneously reduce the side effects caused by chemotherapy (101). The mucositis induced by chemotherapy-induced peripheral neuropathy and intestinal microbial dysbiosis manifests as inflammation from the mouth to the anus and neuropathic pain, respectively (102, 103). These side effects may cause weight loss, generalized infection, and longer hospitalization times (104, 105). Barber et al. (106) demonstrated that taking FO-enriched nutritional supplements could stabilize the appetite and weight of PC patients. The evidence to date demonstrates that ω-3 FAs may decrease cancer risk by affecting genetic variants of inflammatory pathways, oxidative stress, and tumor apoptosis and are also a high potential strategy for the treatment of PC (107, 108). According to the recommendations from the Dietary Guidelines Advisory Committee in 2015, no upper limit was given for dietary fat intake, and saturated fatty acids should be replaced by PUFAs (109). Therefore, sticking to the MD is the right choice to consume adequate PUFAs in a daily diet.

### Micronutrients and PC

Water-soluble vitamins, including folic acid and vitamin C, are well represented in the MD. Vitamin C, also known as ascorbic acid, acts as an antioxidant and is found mainly in citrus fruits, broccoli, spinach, cauliflower, and sweet and white potatoes (110). The combination of hydrogen peroxide and ascorbate in the extracellular fluid has been shown to result in the formation of ROS, which selectively kill tumor cells (111). A study showed that combinations of gemcitabine and vitamin C could inhibit PC tumor growth and demonstrated a gemcitabine dose-sparing effect, even with PC that was unresponsive to gemcitabine (112). Espey (113) and Monti (114) et al. revealed that PDAC patients tolerated the treatment well and showed a decrease in the size of tumors when the intravenous infusion of vitamin C was combined with gemcitabine. It is worth noting that the mechanisms of high-dose intravenous vitamin C are distinct from orally administered vitamin C. Intravenous vitamin C in pharmacologic doses can produce peak plasma concentrations that are several hundredfold higher than those from maximal oral doses (115). A high intake of dietary vitamin C mitigates the risk of PDAC from meat-derived mutagen exposure (116). Another water-soluble vitamin, folic acid, also known as vitamin B9, has a closer relationship with PC compared with other B vitamins. Chittiboyina et al. (117) found that the mean levels of folate in red blood cells were significantly lower in PC patients. Marley et al. (118) demonstrated that dietary folate intake was associated with a reduced PC risk.

Lipid-soluble vitamins are complemented by abundant provitamin A (α- and β-carotene, β-cryptoxanthin) found in yellow-orange-red fruits and vegetables (119). Several meta-analyses have investigated whether dietary vitamin A has an inverse association with PC risk (120, 121). However, the single prospective study showed no association between vitamin A intake and the risk of PC (122). Another lipid-soluble vitamin, vitamin D, is found in oily fish (123). It participates in antitumor effects through the AMPK and PI3K/Akt pathways in PC and inhibits the expression of the cell cycle-related proteins CDKN1A (p21) and CDK1 (124, 125). Altieri et al. (126) found that the immunomodulatory and endocrine regulatory effects of vitamin D are related to the development of diabetes and PC. Camara et al. (127) believed that a lack of sunlight caused vitamin D deficiency, which improved the mortality of PC. Moreover, higher concentrations of vitamin D are suggested to increase the risk of PC (128).

Vitamin E is a group of naturally occurring and potent antioxidants that includes 4 tocopherols and 4 tocotrienols (α-, β-, δ-, and γ-) (129). It has been discovered to inhibit breast cancer, colon cancer, lung cancer, and hepatocellular carcinomas (130, 131). The main sources are vegetable oils and nuts that are frequently consumed in the MD (119). Husain et al. (132) found that tocotrienols inhibit PC by eliminating NF-κB activity. In addition, taking 200 to 1600 mg/day vitamin E δ-tocotrienol before surgery can significantly induce apoptosis in neoplastic cells (133).

Selenium (Se) intake from aquatic and dairy products is seriously insufficient in almost all regions (134). However, large amounts of whole grains, which are good sources of Se, including whole meal pasta and wheat, sourdough bread, stoneground wheat bread, and brown rice, are recommended by the MD (135). Higher levels of Se appeared protective for both mutated and KRAS wild-type PDAC (136). Amaral et al. (137) found that high concentrations of Se were inversely associated with the risk of exocrine PC. Two studies have shown that supplementing Se during cisplatin therapy reduces myelosuppression and nephrotoxicity, suggesting that optimal levels of Se could aid in the toxicity profile related to chemotherapy (138, 139).

Current research on the relationship between these micronutrients and PC does not show a strong association. Hence, a more meaningful approach would be to incorporate certain trace elements into a risk stratification scheme for the selection and surveillance control examination to complement existing PC screening and diagnostic procedures and improve the overall design of future micronutrient clinical trials for PC.

### Polyphenols and PC

Dietary antioxidants counteract oxidation processes and prevent chronic diseases related to oxidative stress (140). Natural antioxidants from plants, including vitamins and phenolic compounds, were suggested to suppress PC, breast cancer, and prostate cancer (141, 142). The phenolic compounds that are closely related to PC include curcumin, resveratrol (RV), and sulforaphane (SFN) (143). Some studies suggest that adding more plant-based condiments, such as curcumin, to the diet could reduce sodium intake (144). Dhillon et al. (145) found that peripheral mononuclear cells isolated from curcumin-treated patients showed reduced activation of NF-κB and that tolerance to gemcitabine and erlotinib was increased. Another clinical trial showed that curcumin improved the MST of gemcitabine-resistant patients and overall survival (146). Li et al. (147) found that SFN could regulate the self-renewal of PC stem cells through the modulation of the Hedgehog pathway. Naumann et al. (148) observed that, when combined with RT, it exerts more distinct DNA damage and pancreatic tumor growth inhibition. In addition, the most striking observation was the ability of SFN to potentiate the activity of several classes of anticancer agents, including paclitaxel, docetaxel, and gemcitabine, through additive and synergistic effects (149). SFN has been explored as a plant-derived histone deacetylase inhibitor in the treatment of PC (150). It is hoped that the novel approach circumvents herculean cancer chemoresistance and alleviates toxicity, the main drawback of monotherapy (151).

RV has been detected in more than 70 plant species, including red grapes, peanuts, and berries (152). It has been shown to target various signaling pathways in PC, such as Hedgehog, leukotriene A4 hydrolase, macrophage inhibitory cytokine-1, and STAT3 (153). Cui et al. (154) demonstrated that RV inhibited the proliferation of PC cells by inducing apoptotic cell death and enhanced the sensitivity to gemcitabine (155). Furthermore, the special effect of RV improves the DNA-damaging effect of paclitaxel in epididymal sperm. This discovery can benefit male cancer patients (156). Verdura et al. (157) demonstrated that the ability of RV to directly target PD-L1 interferes with its stability and trafficking, ultimately impeding its targeting to the cancer cell plasma membrane. However, it did not appear to interfere with blood cell count or splenocyte or macrophage function (158). Available evidence suggests that RV combined with anti-PD-1/PD-L1 blockade treatment can improve the effectiveness of breast cancer immunotherapy (159). This unforeseen immunomodulatory mechanism of RV might illuminate new approaches to restore T-cell function by targeting the PD-1/PD-L1 immunologic checkpoint with natural polyphenols and provide a new perspective for PC immunotherapy.

### Red Meat and PC

Meat intake was considered as a risk factor for PC, but more epidemiological studies should be further explored (160). Some studies believe that its carcinogenicity comes from animal carcinogens, such as heterocyclic amines (HCAs) and benzo(a)pyrene (BaP) (161). However, other studies believe that advanced glycation end products (AGEs) are the main carcinogens. AGEs are a heterogeneous group of compounds present in uncooked foods as well as in food cooked at high temperatures (162). AGEs are associated with insulin resistance, oxidative stress, PC, and chronic inflammation in patients with diabetes (163). AGEs markedly accelerated tumor development in a mouse model of Kras-driven PDAC (164). Jiao et al. (165) observed that higher consumption of red meat increased the risk of PC. Larsson et al. (166) suggested that substituting poultry for red meat might reduce the risk of PC. In addition, some studies refuted the red meat-cancer causality and believed that highly processed meat has a higher risk of causing cancer (167). Thus, future prospective studies should also assess cooking practices and processing methods concerning the risk of PC, not just the type of meat. The serious issue with MD studies is that most research focuses on the prevention rather than the treatment of PC (168). Future studies should shift from using the MD for the prevention of PC to the treatment of PC patients. A recent study proposed a D. I. E. T project to identify the best diet for immunotherapy enhancement against tumors and discussed the dietary patterns affecting immune function. This project proposed adhering to a healthy diet such as the MD, vegetarian, or KD. The proposed supplements are suitable for application in immunotherapy, including ω-3 FAs and polyphenols, which means there is potential for diet in immunotherapy (169).

## The Low-Sugar Diet

Increasing epidemiological evidence has indicated the association between diabetes and pancreatic malignancy, but the mechanism is still unclear (170). Emerging molecular studies suggest that hyperglycemia, obesity-associated hyperinsulinemia, and chronic inflammation in diabetes might be involved in PC proliferation and metastasis (171). The hallmark characteristic of type 2 diabetes (T_2_D) is hyperglycemia, but hyperglycemia can also occur at a pre-T_2_D level with a higher-than-normal blood sugar (BG) level and not reach the threshold for diagnosing T_2_D (172). Hyperglycemic episodes increase the risk of adverse events and outcomes for cancer patients with or without T_2_D (173). Several studies have shown that high glucose activates epidermal growth factor receptor (EGFR), which participates in PC progression (174–176).

The glycemic index (GI) and the glycemic load (GL) were proposed as measurements of carbohydrate quality and quantity (177). A low-GI and an energy-restricted diet containing moderate amounts of carbohydrates may reduce body weight and control glucose and insulin metabolism (178). Those following the low-sugar and low-GI diet should consume large amounts of vegetables rich in fiber and phytonutrients (179), avoid the intake of carbohydrate-rich foods, such as bread, noodles, pasta, and starchy vegetables, as in the Western diet (180, 181), and cakes, candy, biscuits, and sugar-sweetened beverages are forbidden (182). Turati et al. (183) found that high-GL diets may have an adverse effect on blood glucose levels, insulin, and IGFs, resulting in an increased risk of PC. Hu et al. (184) believed that the consumption of sugar, candy, honey, and jam was positively associated with PC. Larsson et al. (185) found that the consumption of added sugar, soft drinks, and sweetened fruit soups or stewed fruit was positively associated with the risk of PC. Other studies have shown that diets high in fructose and sucrose increase the risk of PC, especially for women with a high body mass index or low levels of physical activity (186, 187).

Approximately more than 50% of all patients with PDAC develop diabetes before their cancer diagnosis (188). Diabetes or impaired glucose tolerance is present in more than two-thirds of PC patients (189). The relationship between hyperglycemia, diabetes, and PC is getting closer. Hyperglycemia has been demonstrated to promote the perineural invasion of PC and liver metastasis *in vivo* (190, 191). Kesh et al. (192) observed that microbial dysbiosis caused by hyperglycemia was associated with increased resistance to chemotherapeutic compounds in a T_2_D animal model. Furthermore, high glucose may promote immune escape under a hyperglycemic tumor microenvironment in PC (193). A high-sugar diet brings adverse effects and leads to a poor prognosis. However, some researchers have suggested that a high sugar intake will not increase the risk of PC, with the limitation that patients without diabetes were enrolled in this study (194). Therefore, future studies should explore the relationship between diabetes, abnormal BG, and PC. Meanwhile, a low-sugar diet is more specific and standardized and plays a more targeted role in diabetic patients and people with abnormal BG.

## Conclusion

The existing studies on diet and PC are encouraging, but research is still in its infancy **(** **Table 1** **)**. Research comparing these three diets was even rarer. However, these three diets have a lot in common. The low-sugar diet recommends rejecting refined carbohydrates and excessive sugar intake, which also includes overprocessed meat (180–182). In addition, a low-sugar diet is a low-GI diet, and some researchers believe that the MD is also a low-GI diet (78). In terms of the effect of hyperglycemia on tumors, the mechanism was consistent across these three diets. The mechanism of the KD involves increased oxidative stress and ROS production, which are all related to the hyperglycemic state of the tumor, similar to a low-sugar diet (31–33, 193). Furthermore, views about the types of fatty acids in all three diets were basically the same (15, 16, 21, 80, 81). There have even been studies combining the KD with ω-3 FAs (54).

**Table 1:** Follow-up studies and clinical trials on beneficial diets.

Diet	Country	Year	Tumor types	Combine therapeutics	Phase of trail	Estimated patient enrolment	Primary outcome
**KD**	USA	2012	Advanced Cancer	/	I	10	Patients with stable disease or partial remission had three times higher dietary ketosis than those with continued progressive disease. Preliminary data demonstrated that KD was safe and feasible in selected patients with advanced cancer.
	Korea	2018	PC	Post- pancre atectomy	II	19	Post-pancreatectomy cancer patients who consumed KD had a higher energy intake and body cell mass. That suggested the potential use of KD as an adjuvant anti-cancer therapy.
	Korea	2019	PC	Post- pancre atectomy	II	30	Postoperative KD might beneficially modulated PC-related metabolites in patients with pancreatobiliary cancer. KD might partially provided beneficial effects against PC.
	Turkey	2020	Gastric Cancer	MSCT	II	24	22 patients complete response was achieved. Mean overall survival and mean progression-free survival were prolonged.MSCT appears to be promising in the treatment of advanced GC.
	Turkey	2020	PDAC	MSCT	II	25	Median overall survival and median progression-free survival were prolonged. MSCT was a viable option with the potential to improve survival outcomes of patients diagnosed with metastatic PDAC.
**MD**	Croatia	2003	PC	/	III	100000	The MD could have a protective effect against GC and PC.The standardized incidence rates of PC in areas that adhere to the MD were significantly lower than the average level in other areas.
	Sweden	2012	PC	/	III	77151	Adherence to the MD was inversely proportional to PC mortality. The MD may be associated with chronic disease prevention and better overall health status.
	Italy	2015	PC	/	III	978	This research found that 11.9% of PC were attributable to a low adherence to MD.These results indicated that an appreciable proportion of PC could be avoided by intervening in lifestyle.
	Italy	2016	PC	/	III	2892	Adherence to the MD was negatively related to the risk of PC. These correlations were consistent across strata of age, sex, education, body mass index, alcohol drinking, tobacco smoking and diabetes.
**Low-Sugar Diet**	USA	2002	PC	/	III	88 802	Abnormal glucose metabolism played an important role in pancreatic carcinogenesis. A diet high in glycemic load might increased the risk of PC in women who already have an underlying degree of insulin resistance.
	Sweden	2006	PC	/	III	77797	Frequent consumption of sugar and high-sugar foods might increased the risk of PC by inducing frequent postprandial hyperglycemia, increasing insulin demand and decreasing insulin sensitivity.
	USA	2007	PC	/	III	162150	High fructose and sucrose intakes might play a role in PC etiology. It was more closely related to the risk of PC in people with obesity and insulin resistance.
	Italy	2010	PC	/	III	978	Consumption of sugar, candy, honey, and jam was positively associated with PC. Sweets or refined carbohydrates might increased the risk of PC.
	Italy	2015	Gastrointestinal Cancer et al.	/	III	147090	High-GI and high-GL diets were related to moderately increased risk of cancer at several common sites. Relative risks comparing the highest versus the lowest GI and GL intake were 1.10 and 1.01 for PC.

Although diet has been used to improve immunotherapy and enhance the efficacy of chemotherapy or RT, it is still regarded as a nutritional supplement. Tóth et al. (72–75) have pointed out that nonsurgical therapies might hinder the effects of metabolic therapies and might even lead to the tumor progression in several studies. Therefore, the consideration of using diet therapy as a stand-alone treatment may bring unexpected results. The depth of knowledge about the relationship between diet and cancer is far from sufficient. How these diets bring changes at the cellular level or whether these diets can reduce the risk of PC in the entire family by reducing the risk of the first-generation population and then through genetic variation is still obscure. More clinical trials and more detailed multi-sample studies are needed to better explain the dietary patterns in tumor prevention and treatment. Future research should focus on combining diets with treatment and better popularizing beneficial diets.

## Author Contributions

This review was drafted by YZ and designed by LL. TZ, WY, HZC, XG, GL, HC, and YW conducted the literature search and identified eligible studies. YZ and LL wrote and critically revised the manuscript. BS supervised and reviewed the manuscript. All authors contributed to the article and approved the submitted version.

## Funding

This work was supported by The National Natural Science Foundation of China (81800572, 81670583, 81871974, 81702384).

## Conflict of Interest

The authors declare that the research was conducted in the absence of any commercial or financial relationships that could be construed as a potential conflict of interest.
